# Deletion of *Plasmodium falciparum* Histidine-Rich Protein 2 (*pfhrp2*) and Histidine-Rich Protein 3 (*pfhrp3)* Genes in Colombian Parasites

**DOI:** 10.1371/journal.pone.0131576

**Published:** 2015-07-07

**Authors:** Claribel Murillo Solano, Sheila Akinyi Okoth, Joseph F. Abdallah, Zuleima Pava, Erika Dorado, Sandra Incardona, Curtis S. Huber, Alexandre Macedo de Oliveira, David Bell, Venkatachalam Udhayakumar, John W. Barnwell

**Affiliations:** 1 Centro Internacional de Entrenamiento e Investigaciones Medicas (CIDEIM), Carrera 125 #19–225 Av., La Maria, Cali, Colombia; 2 Malaria Branch, Division of Parasitic Diseases and Malaria, Center for Global Health, Centers for Disease Control and Prevention, 1600 Clifton Road, Atlanta, Georgia, United States of America; 3 Atlanta Research and Education Foundation, Decatur, Georgia, United States of America; 4 Foundation for Innovative New Diagnostics, Chemin des Mines, 1202, Geneva, Switzerland; 5 Global Good Fund/Intellectual Ventures Lab, 1807 132^nd^ Ave NE, Bellevue, Washington, United States of America; Institute of Tropical Medicine, JAPAN

## Abstract

A number of studies have analyzed the performance of malaria rapid diagnostic tests (RDTs) in Colombia with discrepancies in performance being attributed to a combination of factors such as parasite levels, interpretation of RDT results and/or the handling and storage of RDT kits. However, some of the inconsistencies observed with results from *Plasmodium falciparum* histidine-rich protein 2 (PfHRP2)-based RDTs could also be explained by the deletion of the gene that encodes the protein, *pfhrp2*, and its structural homolog, *pfhrp3*, in some parasite isolates. Given that *pfhrp2-* and *pfhrp3*-negative *P*. *falciparum* isolates have been detected in the neighboring Peruvian and Brazilian Amazon regions, we hypothesized that parasites with deletions of *pfhrp2* and *pfhrp3* may also be present in Colombia. In this study we tested 100 historical samples collected between 1999 and 2009 from six Departments in Colombia for the presence of *pfhrp2*, *pfhrp3* and their flanking genes. Seven neutral microsatellites were also used to determine the genetic background of these parasites. In total 18 of 100 parasite isolates were found to have deleted *pfhrp2*, a majority of which (14 of 18) were collected from Amazonas Department, which borders Peru and Brazil. *pfhrp3* deletions were found in 52 of the100 samples collected from all regions of the country. *pfhrp2* flanking genes PF3D7_0831900 and PF3D7_0831700 were deleted in 22 of 100 and in 1 of 100 samples, respectively. *pfhrp3* flanking genes PF3D7_1372100 and PF3D7_1372400 were missing in 55 of 100 and in 57 of 100 samples. Structure analysis of microsatellite data indicated that Colombian samples tested in this study belonged to four clusters and they segregated mostly based on their geographic region. Most of the *pfhrp2*-deleted parasites were assigned to a single cluster and originated from Amazonas Department although a few *pfhrp2*-negative parasites originated from the other three clusters. The presence of a high proportion of *pfhrp2*-negative isolates in the Colombian Amazon may have implications for the use of PfHRP2-based RDTs in the region and may explain inconsistencies observed when PfHRP2-based tests and assays are performed.

## Introduction

Although more than half of the population of Colombia is at risk for malaria infection, malaria in the country has steadily declined from approximately 6 cases per 1000 individuals in 2001 to less than 2 cases per 1000 individuals in 2012 [1]. More than 60% of these malaria cases were caused by *Plasmodium vivax* while the rest were due to *P*. *falciparum* and mixed-species infections [1]. About 79% of all malaria cases (and 71% of all *P*. *falciparum* and mixed species malaria infections) reported in 2011 were concentrated in the Departments of Antioquia, Choco, Cordoba, and Nariño [2]. The fixed-dose combination of artemether and lumefantrine is the first-line treatment for *P*. *falciparum* infections while chloroquine and primaquine are used to treat *P*. *vivax* malaria cases.

In order to administer effective malaria treatment, early and accurate diagnosis is essential. Colombia has a network of diagnostic laboratories and trained microscopists and light microscopy remains the primary method for malaria diagnosis. Nevertheless, malaria rapid diagnostic tests (RDTs) are increasingly being utilized especially in areas where microscopic diagnosis is not feasible [3]. Nowadays, RDT use is not limited to case management in endemic countries; they are also utilized in malaria surveillance and case investigations. Outside malaria endemic countries, RDTs play an important role in disease diagnosis [4]. Pan-specific malaria RDTs target specific antigens, such as lactate dehydrogenase (LDH) and aldolase found in all *Plasmodium* species, while histidine-rich protein 2 (PfHRP2)-based RDTs are specific for *P*. *falciparum*. The majority of the commercially available RDTs target PfHRP2 because of its abundance, high specificity, and thermal stability among other features [5]. A number of PfHRP2-based RDTs cross-react with histidine-rich protein 3 (PfHRP3), a structural homolog with significant sequence similarity and some antigenic cross-reactivity. Factors that affect the accuracy of malaria RDT results include parasite density [6], proper storage and handling of RDT kits [7] and objectivity in interpreting test results [8]. One major concern with RDT performance is the possible false negative results due to the poor quality of tests because they can lead to misdiagnosis and failure to treat. Besides the quality of the tests, other factors such as the absence of the *pfhrp2* gene in natural *P*. *falciparum* isolates can contribute to false negative test results [4]. The first evidence for large scale *pfhrp2* gene deletions in wild *P*. *falciparum* parasites came from Peru [9] and was further substantiated in subsequent studies [10,11]. Other investigations have provided evidence for *pfhrp2* deletion in some parts of Brazil and Suriname [12,13]. Studies conducted in Africa found very low levels of *pfhrp2* deletion especially in Mali, and Senegal but not in the other countries tested [14–16]. One study in India reported very low levels of *pfhrp2* deletion in one state [17] and another study conducted in Honduras failed to find any *pfhrp2* deleted parasites [18]. Evidence for deletion of *pfhrp3*, which is a homologue of *pfhrp2* and contributes to some cross reactivity at the antigen level, has also been documented in several studies [9–13,18].

Given the above reports of *pfhrp2* and *pfhrp3* gene deletions, especially in *P*. *falciparum* parasites from South America, and the false negative test results associated with these deletions, it was important to determine if deletions of *pfhrp2* (and *pfhrp3*) are present in parasites in Colombia. Since the prevalence of *pfhrp2*-negative parasites has been reported to be as high as 40% in the Amazon region of neighboring Peru, [9] and PfHRP2-based malaria RDTs are used for malaria diagnosis in Colombia, it is important to determine if *pfhrp2-* and *pfhrp3*-negative *P*. *falciparum* strains are circulating in Colombia, and contributing to false negative RDT results.

This study investigates the prevalence of *pfhrp2-* and *pfhrp3*-negative *P*. *falciparum* isolates in Colombia using parasite isolates collected at various time points from different parts of the country. We also characterized the parasite isolates genetically so as to determine the genetic background of *pfhrp2*- and *pfhrp3*-negative parasite isolates in relation to their geographical distribution in Colombia.

## Materials and Methods

### Ethics Statement

This study was reviewed and approved by the Institutional Review Board of Colombia’s Centro Internacional de Entrenamiento e Investigaciones (CIDEIM) and the Centers for Disease Control and Prevention (CDC) provided approval for its staff to provide technical support for the study under non-research category. Blood samples were collected once a signed, written consent was obtained from patients. In the case of children, a signed written consent from parents or guardians in addition to an assent from children was obtained prior to sample collection.

### Sample Collection

**Fig 1:**
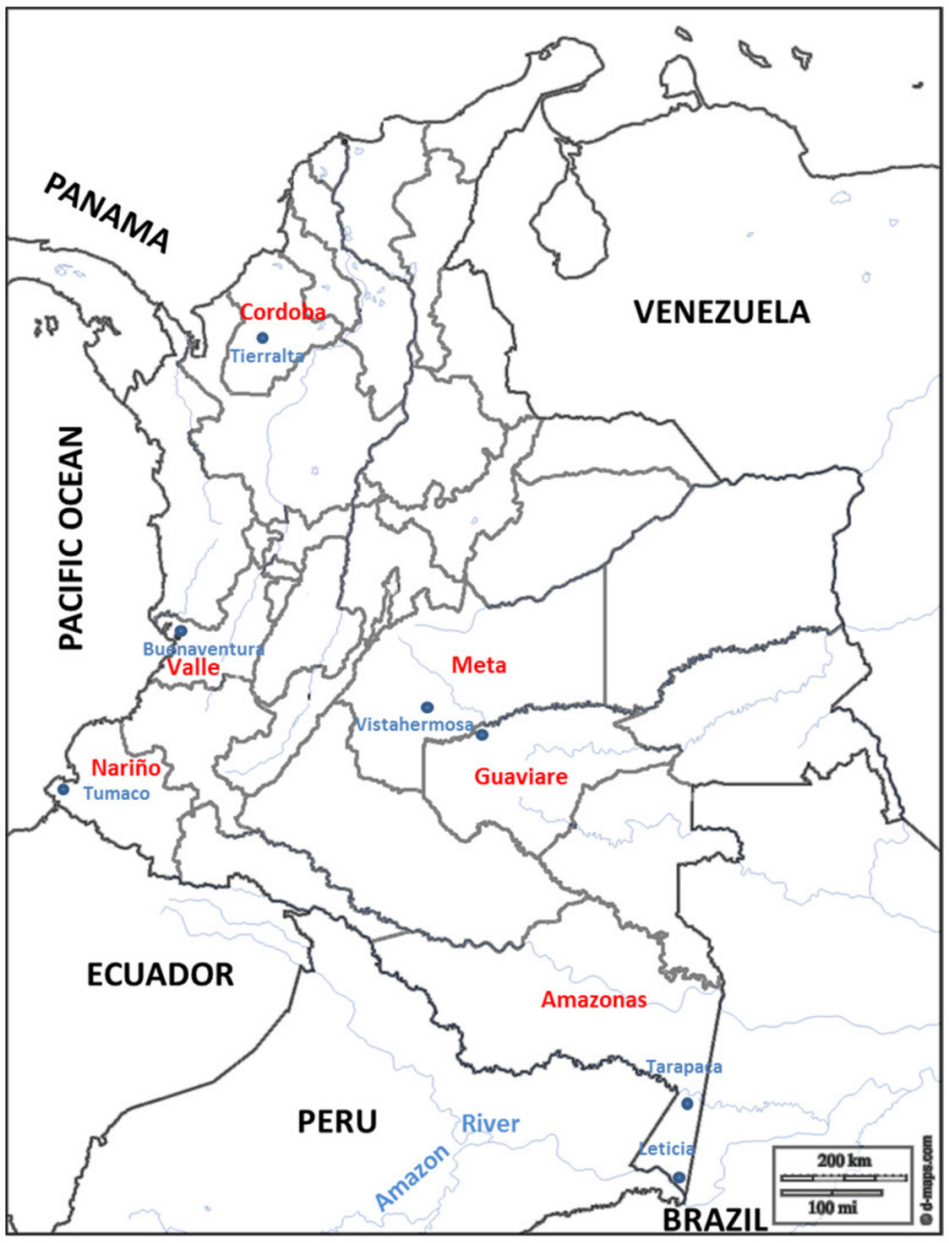
Map of Colombia showing the six departments where patient samples were collected. A total of 115 samples were collected from Nariño (N = 35), Valle del Cauca (N = 35) and Cordoba (N = 20) between 2008 and 2009, and from Amazonas (N = 21), Guaviare (N = 2) and Meta (N = 1) between 1999 and 2007. Blue dots indicate the specific sites where the samples were collected in each department. Map adapted from http://d-maps.com/carte.php?num_car=15145&lang=es.

Dried blood spot samples were prospectively collected from patients with uncomplicated and microscopically confirmed *P*. *falciparum* infections residing in three malaria endemic Departments in Colombia. Blood samples were collected between October 2008 and June 2009 from the Department of Cordoba (N = 20), which is located in northwestern Colombia, and from Nariño (N = 35) and Valle del Cauca (N = 35) Departments, both of which are located along the Pacific West coast. Additionally, filter paper blood spot samples collected from patients residing in the southeastern Departments of Amazonas (N = 21), Guaviare (N = 3) and Meta (N = 1) during epidemiological studies carried out between 1999 and 2007 were also included in this study. Amazonas and Meta Departments report relatively fewer numbers of malaria cases annually [19]. In total, 115 samples collected from six Departments were used (Fig 1).

### Microscopy and Rapid Diagnostic Test Analyses

The 90 blood samples collected from Cordoba, Nariño and Valle were used to prepare Giemsa-stained thick and thin smears and filter paper blood spots (Whatman FTA card and Whatman 3MM, Piscataway, NJ, USA) and for testing with malaria RDTs. After blood collection, all patients received antimalarial treatment according to Colombia’s national guidelines for malaria based on the microscopic diagnosis at the time of presentation (independent of the study results). Thick and thin smears were stained using 5% and 10% Giemsa stain, respectively, for 15 minutes. Stained blood smears were examined using light microscopy by laboratory staff in local clinics and the microscopy results were confirmed by an expert microscopist at CIDEIM. Parasitemia was calculated by counting the number of parasites observed per 200 leukocytes, and assuming a total of 8,000 leukocytes per microliter.

All blood samples were also simultaneously tested using two different malaria RDTs in the field. One test was the PfHRP2-based First Response Malaria Ag HRP2 RDT (Premier Medical Corporation Ltd, Nani Danam, India) and the second was a pLDH-specific Advantage MAL card pLDH (J. Mitra & Co. Pvt Ltd, New Delhi, India). The RDT assays were performed and the results interpreted following the instructions provided by the manufacturers at each site.

### Extraction of Parasite DNA and PCR Analysis

The genomic DNA used for molecular analysis was extracted from filter paper blood spots using the Qiagen DNA extraction kit (QIAGEN, Valencia, California USA). Before conducting molecular analysis for the presence of *pfhrp2*, *pfhrp3* and contiguous flanking genes, each sample was first tested for the successful amplification of merozoite surface protein (*msp)1*, *msp2* and glutamate-rich protein *(glurp)* genes (for samples collected 1999–2007) and *msp2* and *18S rRNA* [20] genes (for samples collected 2008–2009) by nested PCR. Testing for *pfhrp2*, *pfhrp3* and their respective flanking genes were then conducted on samples that met our inclusion criteria of positive amplifications of *msp1*, *msp2*, and *glurp* or *msp2* and *18S rRNA*.

*pfhrp2*, *pfhrp3* and flanking gene amplifications were performed in 20 μl total volume consisting of 10X buffer with 15mM MgCl2, 200 μM dNTPs, 15 μM forward and reverse primers, 0.69 units of Taq Polymerase (Roche, F. Hoffman-LaRoche Ltd, Basel, Switzerland), and 2 μl of DNA template. An *in vitro* cultured *P*. *falciparum* parasite isolate from the Amazon region of Peru was used as a positive control for all *pfhrp2*, *pfhrp3*, and flanking gene amplification experiments. Genomic DNA from cultured Dd2 (5ng/μL) was used as the negative control for all PCR experiments because this isolate lacks *pfhrp2* [21] and its flanking genes [9,11]. Dd2 was also used as a positive control for all the PCR experiments on *pfhrp3* and its flanking genes because it has retained these genes. Similarly, genomic DNA from cultured parasite isolate HB3 (5ng/μL) (A clone from a CDC parasite strain, Honduras I, isolated from a person who travelled to Honduras) was used as the negative control for all *pfhrp3* and flanking gene amplifications because *pfhrp3* [22] and its flanking genes [9] were reported to be absent in this isolate. Additionally, HB3 was used as a positive control for all PCR experiments on *pfhrp2* and its flanking genes because these genes were present in this isolate. Primers and reaction conditions used and expected PCR product sizes have been described previously [18]. All PCR products were separated and visualized on a 2% agarose gel.

For all PCR experiments, a positive gene amplification was recorded as final. When a negative result was obtained, the amplification was repeated for confirmation. If the result from the second amplification was concordant with the first result indicating no gene product amplification, the result was scored as negative. However, if the second result was discordant with the first, the PCR was performed a third time and the two matching results out of three reported as the final result.

### Microsatellite Genotyping and Cluster Analysis

Microsatellite markers were genotyped using whole genome amplified DNA. The whole-genome amplification was performed using 5 μl of genomic DNA per reaction and the Repli-G amplification kit (Qiagen, Valencia, CA, USA). Seven neutral microsatellites were selected for this study so as to determine the population structure of parasites from these sites based on previous studies [23,24]. These microsatellites correspond to the following loci: TA1 and TA109, which are both located on chromosome 6; Poly-α (chromosome 4); PfPK2 (chromosome 12) and 2490 (chromosome 10)[23,25–28]; C2M34 (chromosome 2) and C3M69 (chromosome 3) and amplified using primers and conditions as described [11,29]. The amplification products were labeled with fluorescent dyes (FAM or HEX) and their sizes assayed by capillary electrophoresis on an Applied Biosystems 3130 xl sequencer. The fragments were then scored using GeneMapper software v.3.7 (Applied Biosystems, Foster City, CA, USA) with default microsatellite settings, where allele peaks that were lower than 200 relative fluorescence units (rfu) were defined as background. Allele peaks that were greater than 33% of the height of the predominant allele (which had the highest peak) were scored as additional alleles. When more than one allele was identified in a locus, the sample was deemed to be infected with two or more genetically distinct clones. Samples that did not amplify alleles at some loci on the first attempt were re-tested to get complete data. Only those samples presumed to be infected by a single parasite strain (defined as the amplification of only one allele at each of the seven neutral microsatellite loci) were used for cluster analysis.

To evaluate whether *pfhrp2*- (and *pfhrp3*-) negative isolates had similar genetic backgrounds and if the microsatellite haplotypes we identified clustered according to the geographic origins of the parasite isolates, we applied the Bayesian model-based algorithm implemented in Structure v2.3.3 software [30]. Structure uses a clustering approach to assign isolates to *K* populations characterized by a set of allele frequencies at each locus. We used the same parameters as described previously [11] to test the likelihood of finding between one and ten clusters (*K* = 1 to *K* = 10). We selected these values of *K* based on previous reports of the numbers of distinct sub-populations identified in other South American countries such as Peru [11,24,31] and Colombia [32]. The most likely number of clusters (*K*) was estimated as the value of *K* at the plateau of the mean estimated natural log of the probability estimates of the data [LnP(D)] calculated using the Evanno method [33] and output from the Structure Harvester program [34].

## Results

### Genetic deletion of *pfhrp2*, *pfhrp3* and their flanking genes

Fifteen of the 115 patient samples collected were excluded from genotyping analysis because they failed to amplify one or more of *msp1*, *msp2* and *glurp* or *msp2* and*18s rRNA*. The remaining 100 samples were analyzed for the presence of *pfhrp2*, *pfhrp3* and their respective flanking genes.

**Fig 2:**
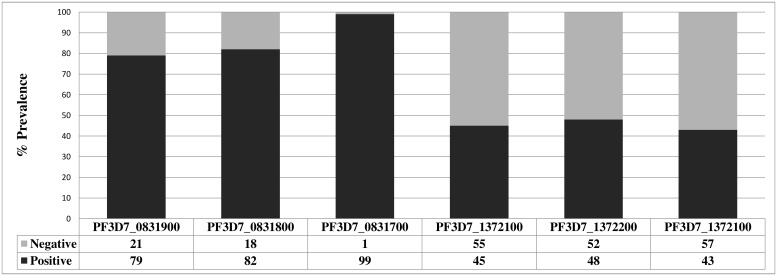
Gene amplification results for *pfhrp2*, *pfhrp3* and their respective flanking genes in *P*. *falciparum* isolates from Colombia.

**Fig 3:**
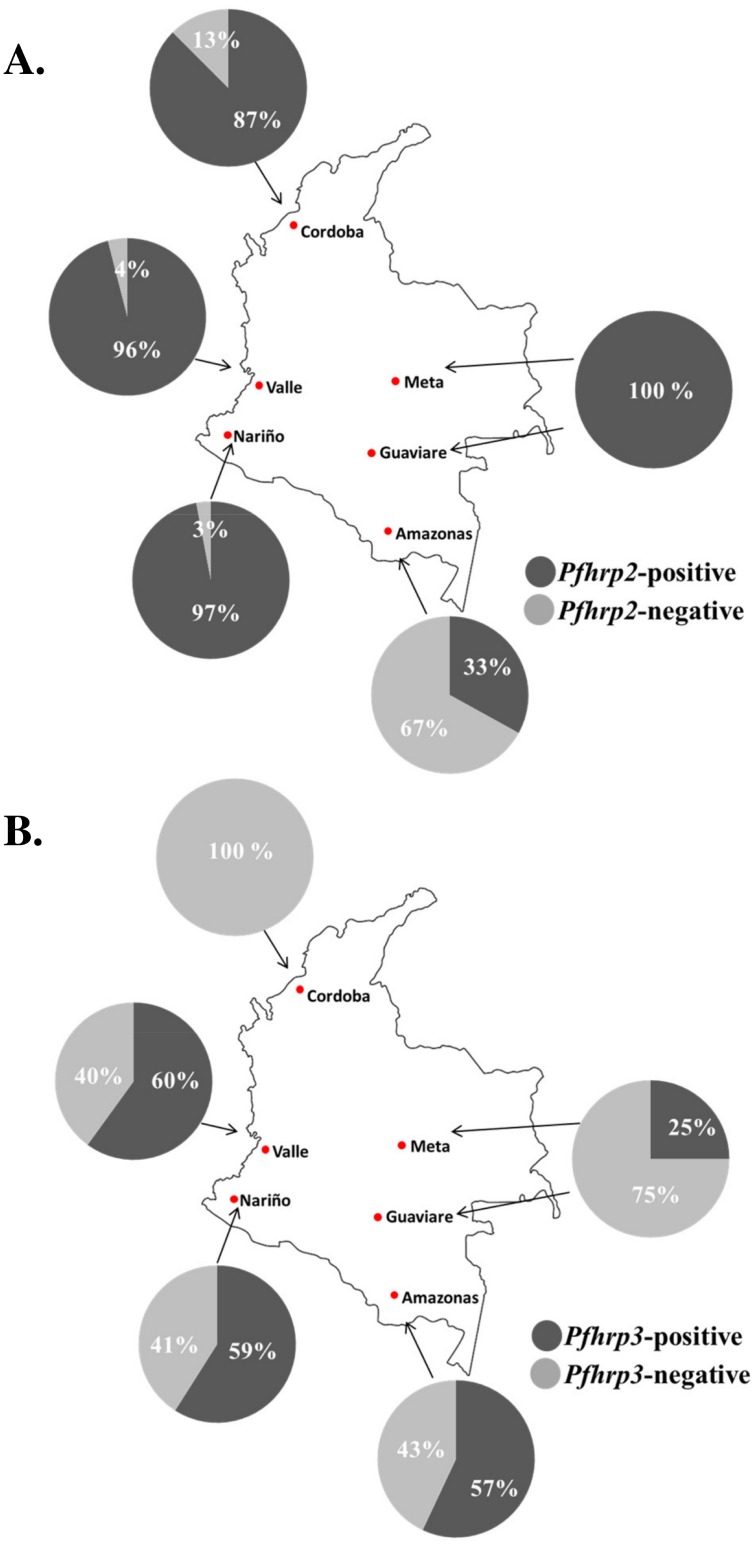
Prevalence of (A) *pfhrp2-* and (B) *pfhrp3*-deleted *P*. *falciparum* isolates in Amazonas (N = 21), Cordoba (N = 16), Valle (N = 25), Nariño (N = 34) and Guaviare-Meta (N = 4).

We found that 18 (18%) of the isolates genotyped were negative for *pfhrp2* while 52 (52%) were lacking *pfhrp3* (Fig 2). A large proportion (14 of 18) of the *pfhrp2*-negative isolates were collected in Amazonas Department while two (sample IDs TA03 and TA08) were from Cordoba, and one isolate each was collected from Nariño (TU005) and Valle del Cauca (BV22) (Fig 3). The highest proportion of *pfhrp3* deletion mutants was found in Cordoba Department where none of the 16 samples tested showed amplification of *pfhrp3* (Fig 3B). In Valle, Nariño, and Amazonas Departments, the proportions of parasites with *pfhrp3* deletions were 40%, 41% and 43%, respectively. In Meta and Guaviare Departments combined, in 3 of the 4 samples tested *pfhrp3* was not amplified.

Overall, 13% of all isolates included deletions of both *pfhrp2* and *pfhrp3* genes (Table 1). Nine of these 13 samples were collected in Amazonas department, while two were from Cordoba, and one each originated from Nariño and Valle. Only five samples were *pfhrp2*-negative and *pfhrp3*-positive, and all five were collected in Amazonas Department.

**Table 1:** Results of PCR amplification of *pfhrp2*, *pfhrp3* and their respective flanking genes in *P*. *falciparum* samples collected in Colombia.

PF3D7_0831900	*pfhrp2*	PF3D7_0831700	PF3D7_1372100	*pfhrp3*	PF3D7_1372400	N
+	+	+	-	-	-	25
+	+	+	+	+	+	16
+	+	+	-	+	+	15
-	+	+	+	+	+	10
+	+	+	+	-	-	8
+	-	+	+	-	-	6
+	-	+	-	-	-	4
-	+	+	-	-	-	4
+	-	+	+	+	-	4
-	-	+	-	-	-	2
-	-	-	-	-	-	1
-	+	+	+	-	-	1
+	+	+	-	+	-	1
-	-	+	-	+	-	1
-	+	+	-	-	+	1
-	+	+	-	+	+	1
**Total**						**100**

Sixteen different *pfhrp2* and *pfhrp3* deletion patterns were observed in our samples (Table 1). A greater proportion of parasite isolates had deleted the gene located 5’ of *pfhrp2*, *PF3D7_0831900*, compared to the 3’ flanking gene, *PF3D7_0831700* (Fig 2). In contrast, there were an almost equal proportion of *PF3D7_1372100* gene deletions (upstream of *pfhrp3*) as there were of *PF3D7_1372400* deletions (downstream of *pfhrp3*) (Fig 2).

### Malaria RDT performance

In order to corroborate the results from our PCR-based genetic deletion analyses, samples collected from Cordoba, Nariño and Valle Departments (N = 90) at the time of blood sampling were tested using a PfHRP2-based RDT. Additionally, pLDH-based RDTs were also used to detect all *P*. *falciparum* parasites regardless of their *pfhrp2* gene deletion status. RDT results for five samples were considered invalid because the control line was reported as negative. Therefore, 85 samples had valid RDT results.

One of the 85 samples (TU009) tested negative with the First Response Malaria Ag HRP2-based test, although the PCR based assay tested positive for *pfhrp2*. However, this specimen was found to have only mature sexual-stage parasites present (Table 2). Three of the 85 samples (TA05, TA10 and TU035) tested negative with the Advantage MAL card pLDH-based RDT (Table 2). TA05 and TA10 had less than 1,000 parasites/μl (Table 2).

**Table 2:** Results for samples for which discordant results were obtained. Pos—Positive result; Neg—Negative result; ND—Sample was not tested.

State of Collection	SampleID	*pfhrp2* PCR	*pfhrp3* PCR	Parasitemia (p/μl)	First Response (PfHRP2)	Advantage MalCard (pLDH)
Cordoba	TA03	Neg	Neg	20,515	Pos	Pos
Cordoba	TA08	Neg	Neg	2743	Pos	Pos
Nariño	TU005	Neg	Neg	168	Pos	Pos
Valle	BV22	Neg	Neg	ND	ND	Pos
Nariño	TU009	Pos	Pos	1120 (only sexual stages)	Neg	Pos
Cordoba	TA05	Pos	Pos	558	Pos	Neg
Cordoba	TA10	Pos	Pos	262	Pos	Neg
Nariño	TU035	Pos	Pos	3675	Pos	Neg

We noted that three of the four isolates collected outside of Amazonas Department (TA03, TA08 and TU005) that were determined to be *pfhrp2* negative by PCR, as well as *pfhrp3* negative, tested positive for the HRP2 protein using the PfHRP2-based RDT kit and also positive for pLDH using the Advantage MalCard RDT (Table 2).

### Cluster Analysis

**Fig 4:**
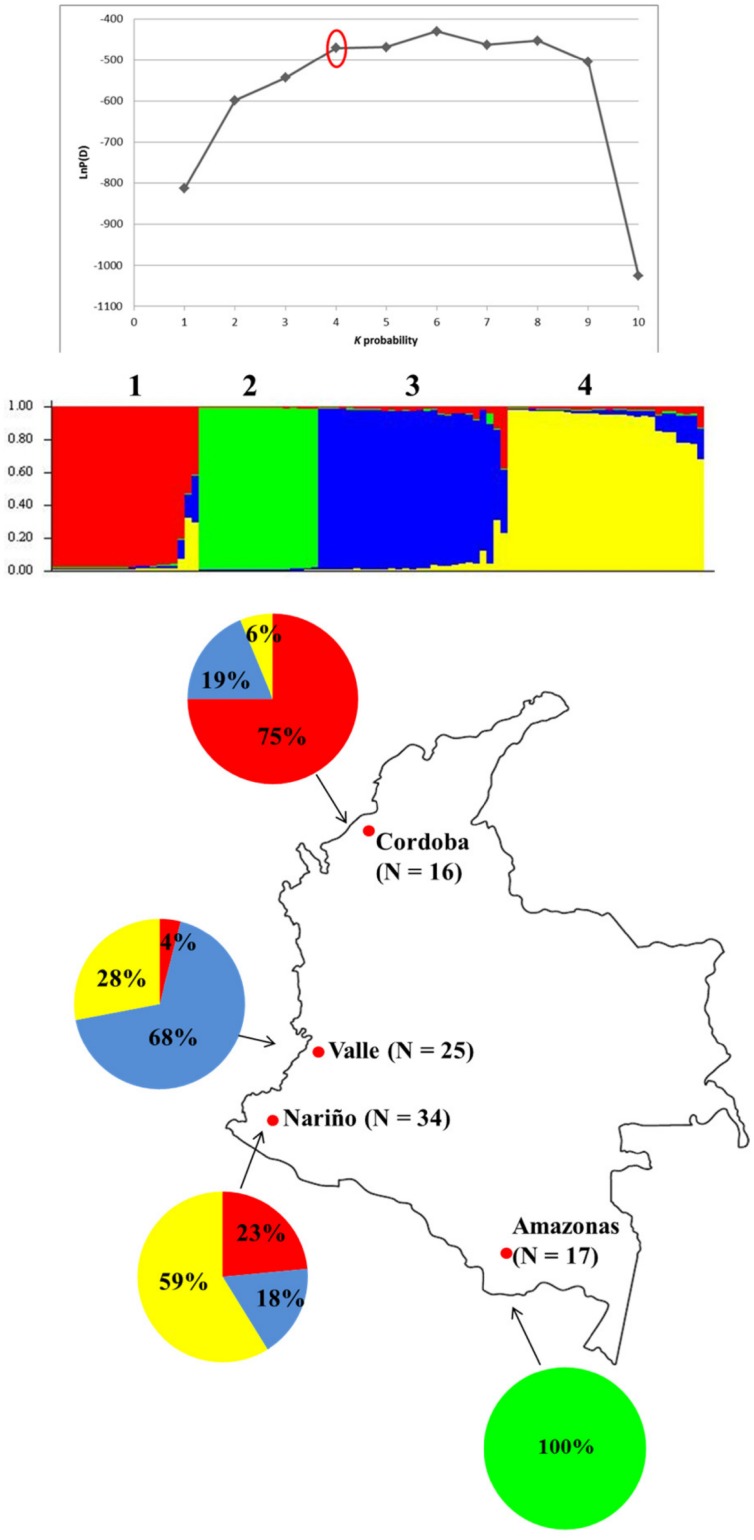
Population structure of *P*. *falciparum*-infected samples from Colombia (N = 104). (A) Plot of Var[LnP(D) vs. *K* probability showing the best *K* subpopulation partition at *K* = 4. (B) Output from Structure v2.3.3. Each color corresponds to a population (labeled 1 to 4) classified by Structure. Each individual isolate is represented by a vertical bar. The Y axis represents the estimated proportion of membership of an individual to each predicted population cluster. The number of isolates assigned to each cluster were as follows: Cluster 1 (N = 21); 2 (N = 17); 3 (N = 26); 4 (N = 28). (c). Distribution of the four clusters across the four departments. Red—Cluster 1; Green—Cluster 2; Blue—Cluster 3; Yellow—Cluster 4. The total number of samples analyzed from each department is shown in parentheses.

We genotyped seven neutral microsatellite loci in singly infected isolates that met our inclusion criteria (N = 92) and performed cluster analysis to investigate the association between the geographic origins of the *P*. *falciparum* isolates, their *pfhrp2*/*pfhrp3* deletion status and their membership in the groups predicted by Structure software. Cluster analysis inferred population substructure in this group of isolates and the number of population clusters (*K*) was predicted to be four (Fig 4).

Generally, the parasite isolates appeared to cluster based on their in-country geographic origins. All 17 isolates (100%) assigned to cluster 2 originated only from Amazonas Department (Fig 4B and 4C). More than half (59%) of the 34 parasite isolates collected in Nariño Department could be assigned to cluster 4, while 17 isolates or 68% of those specimens collected in Valle were assigned to cluster 3 (Fig 4B and 4C). Three quarters of the isolates (12) from Cordoba were assigned to cluster 1 (Fig 4B and 4C).

The distribution of *pfhrp2-* and *pfhrp3*-negative isolates among the clusters was skewed; we observed that 12 of the 18 *pfhrp2*-negative isolates (67%) were assigned to cluster 2, which consisted solely of samples from Amazonas Department (Figs 3A and 4B). Three other *pfhrp2*-negative isolates were each assigned to clusters 1, 3 and 4. Moreover, eight of the 13 *pfhrp2*/*pfhrp3*-double negative isolates (61.5%) were also designated to cluster 2. Whereas Structure analysis of the neutral microsatellite allele lengths for samples in cluster 2 predicted the membership of these samples solely to that cluster, some samples assigned to clusters 1, 3 and 4 appeared to have varying levels of admixture indicated by multi-colored bars which showed their relative probability of assignment to more than one cluster (Fig 4B).

## Discussion

Overall, this study has shown that *pfhrp2*-deleted parasites are highly prevalent in Amazonas department, but rarely found in the coastal sites that were examined. This finding suggests that PfHRP2-based RDTs are likely to detect most *P*. *falciparum* parasites in Cordoba, Valle del Cauca and Nariño, but not in Amazonas Department, where we detected a 67% deletion rate for *pfhrp2* among the limited number of *P*. *falciparum* isolates analyzed. This high prevalence of *pfhrp2* (and *pfhrp3*) deletions in samples from Amazonas, was considerably higher compared to the other sites surveyed in Colombia (67% vs 5.0%, respectively). This deletion rate of 5% for *pfhrp2* may actually be lower as three of the four non-Amazonas isolates that tested negative for *pfhrp2* by PCR were initially positive by an HRP2-based RDT (see below). The presence of parasites with *pfhrp2* deletions in Colombia, especially in Amazonas, supports the previous observations that these deletions are more frequent in South America [9–13] compared with other regions where this phenomenon has been investigated [14–17].

Our results also show that *pfhrp3* deletions are more prevalent than *pfhrp2* deletions in Colombia, which also has been found to be the case in Peru and Honduras [9,11,18]. Interestingly, this is not the case in Suriname where *pfhrp2*-negative parasites were detected in higher numbers than *pfhrp3*-negative isolates [13]. Whereas *pfhrp2*-negative parasites were mostly limited to the Amazon region located on the border with Peru, *pfhrp3*-negative isolates were distributed across all six departments surveyed. Overall, outside of Amazonas the prevalence of *pfhrp3* deletions was 54% compared to 43% within the Department of Amazonas.

Two important questions that arise are why there are multiple patterns of deletion within the region of *pfhrp2* and *pfhrp3* flanking genes and if there is an orderly pattern for such deletions. It is evident from a previously published study using tiling array that deletion of parts of chromosomal segments within 20 kb of both *pfhrp2* and *pfhrp3* occurs in natural *P*. *falciparum* isolates obtained from Peru [35]. The patterns of deletion varied among the limited isolates studied. In terms of physical location, *pfhrp3* is proximal to the end of the chromosome 13 while *pfhrp2* is located in the subtelomeric region of chromosome 8. It has been proposed that some subtelomeric regions of the chromosome of *P*. *falciparum* laboratory and field isolates are the target of deletional activity which is then accompanied by repair mechanisms via enzymes such as telomerases [36–39]. Thus, it is not clear at this time if *pfhrp2* and/or *pfhrp3* deletion are driven by any specific mechanism or if they are due to some broad chromosomal deletion events occurring in selected regions of the genome where these genes are located. Clearly, further studies using next generation sequencing methods are needed to answer mechanisms associated with these genetic deletions.

The results from this study may provide some context to a recent report from Colombia in which the *in vitro* susceptibility of *P*. *falciparum* parasites to various antimalarial drugs was tested [40]. The study utilized schizont maturation and PfHRP2-ELISA methods as read-outs for their assays [40]. It was determined that PfHRP2 could not be detected by ELISA in sixteen out of twenty samples (65%) collected in Leticia (a site located in Amazonas Department) even though the schizont maturation method was able to yield results with these samples [40]. This observation is consistent with current findings indicating deletion of *pfhrp2* gene in a majority of samples (67%) in the Amazonas Department.

The results from microsatellite-based genetic diversity data indicate that the *P*. *falciparum* population in Colombia has undergone a bottleneck or bottlenecks event sometime in the recent past. Overall, the parasite population in Colombia from analysis of our genetic data appears to be comprised of four major clusters. This finding is consistent with a recent SNP study in Colombia that showed the presence of four clusters of *P*. *falciparum* parasites in samples collected from four departments located along Colombia’s Pacific coast (Cauca, Chocó, Valle and Nariño) [32]. Geographic clustering was also apparent in our sample set; one of these four clusters consisted only of isolates collected from Amazonas Department while the majority of isolates in each of the other three clusters originated from specific Departments, Nariño, Valle and Cordoba. Importantly, most of the *pfhrp2* deleted parasites were assigned to cluster 2, which consisted of isolates from Amazonas Department. It is likely that this may be due to the clonal expansion of a *pfhrp2*-deleted lineage. Overall, these findings in Colombia are similar to those from Peru where all deletion*s of pfhrp2* were found in different frequencies in various geographical locations within the Amazon River basin of Peru. As noted above, given that the Department of Amazonas in Colombia is contiguous with the same ecological zone in Peru, it is possible that the *pfhrp2*-negative isolates in Colombia are genetically related to some of the *pfhrp2*-negative parasite isolates circulating in the Peruvian Amazon. Even though the prevalence of *pfhrp2*-negative parasites is low outside of Amazonas Department, migration events could lead to their spread to other departments in Colombia as was the case for some sulphadoxine-pyrimethamine- resistant parasites [41].

It should be noted, however, that these microsatellite analyses were only performed on samples from Cordoba, Valle, Nariño and Amazonas. Therefore, in order to better elucidate the effect of geographic origin on allele diversity, future studies would require analysis of samples collected from other Departments such as Antioquia, Cauca and Chocó, which have relatively higher prevalence of *P*. *falciparum* malaria [19]. In addition, given the results obtained from this study, a larger sampling from regions such as Guaviare and Meta will likely provide greater insights.

We did note some discrepancies in results between the PfHRP2-based RDTs and PCR genotyping for three samples, where the PCR-based genotyping produced a negative result for *pfhrp2* while a PfHRP2-based RDT produced positive results. One interpretation of this result is that the PCR test was not able to detect the *Pfhrp2* gene that were indeed present, as evidenced by the positive HRP2-based RDT, in these three samples, which originated from the coastal region. This possible failure in the PCR could have occurred if mutations in the priming site complementary to the 3’ end of the PCR primer were present in the samples. The high polymorphism in the *pfhrp2* gene found in clinical isolates could support this hypothesis [42]. Another possible explanation is that the results of the PfHRP2-based and pLDH- based RDTs might have been interpreted incorrectly or gave a false positive result, such as has been noted in the past for PfHRP2-based RDTS tested with blood specimens containing with rheumatoid factor (RF). However, PfHRP2-based RDT used in this study is one of the top performing commercial malaria tests with a very high specificity, which makes the occurrence of false positives at this frequency very unlikely. Also, Advantage MalCard pLDH-based RDT were positive, reducing the possibility of the false positive due to RDT factors. Detecting genetic deletions of *pfhrp2* and *pfhrp3* using PCR requires caution because the absence of a PCR amplified product is being investigated. There are only three short conserved regions in the *pfhrp2* and *pfhrp3* genes from where primers can be selected, which hampers optimizing for the most robust amplifications possible. And therefore, any loss of DNA through partial degradation or lower parasitemia will compromise obtaining true negative results. In our experience, DNA isolated directly from blood performs significantly better for amplifying the *pfhrp2* (or *pfhrp3*) gene compared to DNA isolated from blood spotted on filter paper, especially, if the paper is not of highest quality and not stored dessicated at or below 4°C. For logistical reasons, only filter paper blood spots were available for this study, and it is therefore possible that this may have contributed to these discordant results. It is very difficult to rule out false negative test results using PCR data alone; complementing gene amplification data with PfHRP2 protein detection for confirmation is ideal. Since we did not have plasma samples available, ELISA confirmatory testing could not be performed. On the other hand, RDT testing can provide additional supporting data, although most samples but not all samples were tested with RDTs in this study.

This study shows that *pfhrp2*-negative *P*. *falciparum* isolates are present in Colombia, mostly in the Amazon region. Regular monitoring for this parasite population will inform national policy on the utilization of PfHRP2-based RDTs for malaria detection in areas where microscopy is not available. Nevertheless, this study had some limitations. For one, samples were collected only from some of the Departments where *P*. *falciparum* occurs in Colombia and our findings cannot necessarily be extrapolated to all parts of the country. Moreover, sampling and the type of specimens obtained were done based on logistical convenience and the number of samples from some sites was limited. Furthermore, a proportion of the samples reported here were collected between 1999 and 2007 for other study purposes. A larger and more systematic collection and analysis of parasite isolates from Amazonas and other parts of the country, whether along the coastal regions or in the interior, will clarify the current prevalence and distribution of *P*. *falciparum* isolates with *pfhrp2* and *pfhrp3* deletions in the country overall.
